# P43 *In vitro* activity of cefiderocol and comparators against Gram-negative pathogens: ARTEMIS study in the UK

**DOI:** 10.1093/jacamr/dlac004.042

**Published:** 2022-02-16

**Authors:** D. Wareham, M. Attwood, A. L. Casey, M. Coyne, D. Hughes, M. Lister, J. Nahl, J. D. Perry, A. Santerre Henriksen, C. Longshaw

**Affiliations:** Queen Mary University, London, UK; PK/PD Laboratory, North Bristol NHS Trust, UK; University Hospitals Birmingham NHS Foundation Trust, Birmingham, UK; Scottish Micro Ref Lab (SMiRL), Glasgow, UK; Manchester University Hospital NHS Foundation Trust, Manchester, UK; Nottingham University Hospitals NHS Trust, Nottingham, UK; Leeds Teaching Hospitals NHS Trust, Leeds, UK; Freeman Hospital, Newcastle upon Tyne, UK; Maxel Consulting ApS, Jyllinge, Denmark; contractor for Shionogi; Infectious Diseases, Shionogi B.V., London, UK

## Abstract

**Objectives:**

Cefiderocol (CFDC) is a novel siderophore cephalosporin approved in Europe for the treatment of infections caused by aerobic Gram-negative (GN) bacteria in adults with limited treatment options. The aim of the ARTEMIS study was to evaluate the *in vitro* activity of CFDC and comparators against recent clinical isolates collected across five countries in Europe. Here we report susceptibility data from isolates collected in the UK.

**Methods:**

From January to December 2020, GN clinical isolates were collected from hospitalized patients from all infection sites (excluding the urinary tract). Duplicate isolates of the same species from a single patient were excluded. As a prespecified target, each laboratory collected 75 isolates, with: 20 *Klebsiella* spp., 20 other Enterobacterales, 20 *Pseudomonas aeruginosa* and 15 *Acinetobacter baumannii* isolates expected to be included. CFDC susceptibility testing was conducted using disc diffusion (with 30 μg discs) on Mueller–Hinton agar and Sensititre^™^ broth microdilution (BMD) panels [EUMDROXF; centrally tested at International Health Management Associates (IHMA)]. Susceptibility by disc diffusion was reported using zone diameter breakpoints (BPs) of ≥22 mm (or ≥17 mm for *A. baumannii* isolates, corresponding to MIC values below the pharmacokinetic/pharmacodynamic BPs of ≤2 mg/L). Comparator susceptibility was determined using custom research use only Sensititre^™^ BMD panels (CMP2SHIH) according to the EUCAST method for BMD. Antimicrobial susceptibility was interpreted according to EUCAST clinical BPs (v.11 2021).

**Results:**

In total, 517 isolates were collected from nine UK hospitals, of which: 308 (59.6%) were Enterobacterales [including 147 (28.4%) *Klebsiella* spp.], 148 (28.6%) were *P. aeruginosa* and 33 (6.4%) were *A. baumannii*. The most common sites of infection were bloodstream (*n = *245; 47.4%), respiratory tract (*n = *158; 30.6%) and skin (*n = *59; 11.4%). A high percentage of Enterobacterales (90.2%), *P. aeruginosa* (96.6%) and *A. baumannii* (96.9%) isolates were susceptible to CFDC by disc diffusion. By central laboratory testing (MIC), 99.0% of Enterobacterales, 99.3% of *P. aeruginosa* and 93.9% of *A. baumannii* isolates were susceptible to CFDC. High susceptibility rates (>85%) were also observed for all comparator agents (Table 1). A total of 32/517 (6.2%) isolates were carbapenem resistant, the majority of which (22/32, 68.8%) were susceptible to CFDC by disc diffusion.

**Conclusions:**

Among clinical GN isolates collected from UK hospitals in 2020, a high percentage (98.6%), including carbapenem-resistant isolates, were susceptible to CFDC by BMD. These data support the use of CFDC in patients with GN infections and limited treatment options. The differences identified between EUCAST disc diffusion and BMD using Sensititre^™^ panels for CFDC highlight that disc diffusion underestimates Enterobacterales susceptibility to CFDC, which is mainly a result of the area of technical uncertainty (where isolates with MIC of 2 mg/L have a zone diameter of <22 mm and are characterized as resistant). This requires further investigation to explore whether the EUCAST zone diameter BP is optimal for CFDC disc testing.

**Table 1.** CFDC susceptibility based on disc zone diameters; CFDC and comparator agent susceptibility based on MIC from BMD (EUCAST clinical BPs v.11, 2021)

Susceptibility, *n*/*N* (%)	CFDC (disc)	No. isolates in ATU	CFDC (MIC)	MEM^a^	C/T	CZA	MVB	I/R	CST
Enterobacterales	277/307 (90.2)	40/307 (13.0)	285/288 (99.0)	295/308 (95.8)	264/308 (85.7)	298/307 (97.1)	303/308 (98.4)	286/306 (93.5)	265/307 (86.3)
*P. aeruginosa*	143/148 (96.6)	8/148 (5.4)	144/145 (99.3)	135/148 (91.2)	140/148 (94.6)	141/148 (95.3)	137/148 (92.6)	133/147 (90.5)	142/148 (95.9)
*A. baumannii*	31/32 (96.9)	N/A	31/33 (93.9)	30/33 (90.9)	N/A	N/A	N/A	30/33 (90.9)	30/33 (90.9)

ATU, area of technical uncertainty; CFDC, cefiderocol; CST, colistin; C/T, ceftolozane/tazobactam; CZA, ceftazidime/avibactam; I/R, imipenem/relebactam; MEM, meropenem; MVB, meropenem/vaborbactam.

a Isolates were defined as MEM-susceptible with a BP of ≤8 mg/L [relating to high-dose extended-infusion (2 g, 3 h infusion) MEM], based on EUCAST recommendations.

